# Adult children of parents with mental illness: parenting journeys

**DOI:** 10.1186/s40359-018-0248-x

**Published:** 2018-07-27

**Authors:** Gillian Murphy, Kath Peters, Lesley Wilkes, Debra Jackson

**Affiliations:** 1 School of Nursing and Midwifery, Translational Health Research Institute, Western Sydney University, Penrith, NSW 2751 Australia; 2 School of Nursing and Midwifery, Western Sydney University, Penrith, NSW 2751 Australia; 3 Centre for Nursing Research and Practice Development, Nepean Blue Mountains Local Health District, First Floor, Court Building, Nepean Hospital, PO Box 63, Penrith, NSW 2751 Australia; 4 Faculty of Health, University of Technology Sydney, Ultimo Campus, Sydney, Australia; 5 University of New England, Armidale, New South Wales Australia

**Keywords:** Anxiety, Childhood, Family relations, Intergenerational relationships, Parental mental illness, Mental health and parenting

## Abstract

**Background:**

Individuals who have lived with childhood parental mental illness are at increased risk of developing mental health concerns. Yet there is limited knowledge about how a person’s childhood experiences of parental mental illness may influence their subsequent parenting roles.

**Methods:**

This narrative study generated parenting narratives of adult children who had lived with childhood parental mental illness. Interviewees included 10 women and three men. Inductive thematic analysis was used to establish themes and sub-themes from the narratives.

**Results:**

The theme of parenting journeys with sub-themes of: ‘adult children living with parenting worries’ and ‘adult children seeking emotional connectivity with their children and others’ are presented.

**Conclusions:**

Parenting anxiety may be a common experience shared by all parents. However, adult children’s worries in relation to their child/ren developing mental illness may be associated with their own experiences of childhood parental mental illness. All health professionals have a pinnacle role in supporting families to build resilience and harness positive experiences within familial relationships to recognise and mitigate parenting anxiety.

## Background

Children of parents with mental illness present with greater prevalence of mental health concerns [1–3]. A meta-synthesis of 22 studies dated 2000 to 2012 found children experienced unpredictable daily life with an increased sense of responsibility, blame and worry [4]. Additionally, children reported stigma and a need to keep secrets, while wanting greater information regarding their parent’s mental illness [4]. These findings were further supported by a systematic review using eight studies in 2018, which suggested that children sought a greater understanding of mental illness and but also noted that children had concerns about their relationships with their parents [5]. Access to preventative programmes for children with parents with mental illness was difficult, due to barriers including: children’s reluctance to engage as a result of perceived and actual stigma; children’s fear of information disclosure to their parents; limited programme provision; lack of health professional’s discussions with parents and children regarding a child’s needs and logistics such as transport and finance [3]. Despite this, there continues to be limited primary health care service provision to support the needs of this group of children and young people. Additionally, mental health services have not as yet, adopted a whole of family care ethos [6, 7]. Given the limited access to service provision, a child’s experiences of fear and distress associated with living with a parent with mental illness may continue into their early adult life.

Young adults who lived with childhood parental mental illness are thought to experience a similar sense of increased responsibility with loneliness and isolation; noting feelings of difference to others, while living in a highly emotional home environment [8]. Recent study findings [8] were supported by Petrowki & Stein [9] who highlighted that some, but not all interviewees, experienced “negative relationships” with their parent with mental illness ([9], p., 2877). The experiences of children and young adults were consistent with findings offered by Foster [10] who found that adults who lived with childhood parental mental illness had “feelings of uncertainty” and “struggled to connect” with others [p. 3143].

Despite the consistent findings of concerning experiences for children and adults who have lived with childhood parental mental illness, individuals suggested they were hopeful of positive change as they actively sought balance ([10], p., 3143). Familial resilience building was helpful, as were strategies such as daily routines and humour [11]. Yet, some studies demonstrated that children who are living with, and adults who have lived with parents with mental illness required a greater understanding of mental illness [11, 12], demonstrating a potential role for all health and social care professionals to support and educate families. Despite the body of knowledge regarding child and adult experiences of childhood parental mental illness, there have been calls to enhance discussions beyond risk and resilience, leading to a greater emphasis of the lived experiences of families [13]. We argue there remains a paucity of research giving voice to both children’s and adult children’s experiences of living with a parent with mental illness and one’s subsequent parenting role.

### Aims of the paper

This paper is drawn from a larger doctoral study which sought to generate the experiences of adult children who had lived with childhood parental mental illness and their subsequent parenting roles. Findings on living with fear and mistrust [14]; navigating stigma [15]; loss of self [16] and dehumanisation of a parent with mental illness [17] were previously published. This current paper reports findings on the subsequent parenting journeys of adults who lived with childhood parental mental illness.

## Methods

### Narrative inquiry

The study employed a narrative approach. Narratives are used in a range of educational environments and therapeutic health settings [18–20]. Moreover, the use of narratives in research is a common phenomenon [21–23]. When used in research, narratives enhance understanding and provide differing perspectives which ultimately add to the “humanization of care” ([24], p. 1) in clinical practice areas. Additionally, narratives help to contextualise values and perceptions within cultural and societal expectations [23]. While narratives are utilised in research to gather rich information regarding specific areas of interest, they can also be incredibly powerful to enhance interviewee’s understanding and perceptions of their own lived experiences [14, 19].

Trustworthiness of narrative data can be achieved utilising researcher reflections during study design, recruitment, data collection, analysis and reporting. Integrity and trustworthiness of the study was confirmed using the 32 item COREQ (COnsolidated criteria for REporting Qualitative research) checklist throughout the research process [25]. The COREQ helps to clarify the integrity of research studies specifically using interviews and focus groups [25].

## Recruitment

Participant recruitment was undertaken for this doctoral study from 2009 to 2016. In keeping with the ethical approval from a University Human Ethics Committee, potential interviewees were invited into the study using local media (newspapers and radio) in an East Australian city. Additionally, flyers were sent to local neighbourhood centres for the attention of community members. In keeping with purposive sampling, the inclusion criteria were included in both the media release and study flyers. Potential interviewees were asked to make initial contact with the lead author for additional information and to undertake screening questions to clarify their suitability for the study. The general screening questions clarified that potential interviewees met all of the study inclusion criteria. Study information and consent forms were posted or mailed to potential interviewees, dependent on individual preference. Potential interviewees were asked to make further contact with the researcher if they wanted to take part in the study. This provided each person with time and space to consider their involvement with the study given the sensitive nature of the research, enhancing the integrity of informed consent.

### Inclusion criteria

Inclusion criteria ensured all interviewees were suitable for the aim of the study. Inclusion criteria included: interviewees were at least 18 years of age; English speaking; able to be interviewed in person, over the telephone or live exchange email; experienced a parent diagnosed with psychosis, psychosis related disorder, serious thought or mood disorder during their childhood; their parent had been hospitalised for mental health treatment during their childhood and must be a parent with no past or current mental health treatment themselves.

### Data collection

Ten women and three men, ranging from 30 to 78 years old participated in the study. Individual interviewees met with the lead author for 1 h to 90 min at a mutually agreed time. Meetings with individual interviewees took place in private rooms on university campuses, in a community centre or library. Semi structured interviews were used to facilitate interviewee’s reflections about their experiences within an emotionally safe environment. The researcher supported the interviewees to construct their narratives using two main open-ended questions: “what were your experiences of being parented by a person with mental illness?” and “what were your own parenting experiences?” Subsequent funnelling and focussing questions were asked throughout the meeting, to gather detailed information of interviewee’s experiences, such as: “how did that particular experience influence your time with other family members?” and “how did that experience make you feel?”. All meetings were digitally recorded and transcribed into written form with interviewee consent. All transcripts were de-identified for analysis.

### Analysis

The authors used an inductive, thematic analytical approach to establish the study findings. Thematic analysis is a common approach to identify areas of interest and themes within qualitative data [26]. All transcripts were initially reviewed to establish a general understanding of interviewee’s experiences. Individual transcripts were then considered by the main study researcher to generate themes and sub-themes. Subsequently, three other members of the research team considered the transcripts, themes and sub-themes originally noted by the main study researcher to clarify and confirm interpretations. Establishing study trustworthiness is central to qualitative data analysis to ensure data and findings are interpreted and presented accurately [27]. The research team, consisted of four members, GM, KP, LW and DJ, who met regularly during the data collection and analytical process to ensure consistency of data interpretation and study integrity. Discussions about themes and sub-themes continued until there was a group consensus. On-going team discussions about data interpretation and the development of themes and sub-themes from the data helped to mitigate individual researcher biases. It was apparent during the initial analytical process, that the interviewees made significant reference to their teenage years. Given this, transcripts were reviewed again to generate a greater understanding of significant times in the interviewee’s life’s. Childhood, teenager, young adult and parenthood periods were considered. When the individual transcripts were organised as themes, they were compared to establish suitable themes and sub-themes for the community of adult children who had been involved in the study. In essence, a community story incorporating all of the adult children’s narratives was generated.

## Results

The theme of adult children’s parenting experiences and associated sub-themes: adult children living with parenting worries and adult children seeking connectivity with their children and others are presented in this paper. See Table 1 for details of the findings associated with this particular theme and sub-themes.

**Table 1:** - Sub-themes

Sub-themes	Categories	Main coding from transcripts
Adult children living with parenting worries	Worries about children’s emotional health	Worries about children developing mental illness. Hyper-vigilante to child’s emotional health. Finding the children’s emotions difficult to understand.
	Parenting challenges	Finding communications with child challenging. Struggling to develop emotional connectivity with child.
	Parenting self-doubts	Self-doubt and questioning parenting – lacking confidence. Difficulties ensuring consistency with children. Have to consciously think about parenting more than others.
	Lacking an internal parenting framework and support	Lacking a parenting framework and role model. Lacking guidance in parenting role.
	Oppositional parenting	Fears of making the same mistakes as own parents. Using an opposite approach to parenting.
Adult children seeking connectivity with their children and others	Wanting better and the best for the children	Want success, happiness, community role for children. Wanting something different for children than own (adult children’s) experiences. Wanting to be the best parent possible.
	Wanting love and connectivity with children	Importance of being with family and children. Being involved with children’s daily lives and enjoying family time. Want to give children so much love – being loving, respectful, open and developing trust.
	Wanting to protect or be protected by children	Want to protect children. Not wanting children to go through the same as adult children.
	Wanting to provide space for children to grow	Allow children to follow their desires – provide opportunities for them. Independence is key. Actively developing resilience in children.

### Adult children living with parenting worries

Anxiety was identified as a common experience for many interviewees during their own childhoods while living with their parent with mental illness. Additionally, 10 out of the 13 interviewees made explicit reference to worry and anxieties within their own parenting roles. Several of the interviewees highlighted anxiety about the possibility that their own children may be at increased risk of mental illness, given their parent’s diagnosis of mood disorder or psychotic related illnesses. Interviewee two whose mother had a diagnosis of schizophrenia, expressed concerns about a son when stating:

*“Sometimes I wonder if they are going to get a mental illness.”*

Another interviewee (13) had taken action to warn the children of the risks of mental illness. Interestingly, the interviewee felt responsible for the children’s possible genetic susceptibility, despite alluding to one’s own childhood experiences:

*“I’ve talked to all of our children about the risks that they face. I’ve warned them particularly of drugs and cannabis, not because they are what they are, but because of their probable susceptibility. Look, I could be wrong. I could have empowered them with the potential for illness in a completely irresponsible way. These things are about numbers. They’re about genetic inheritance. I’ve taken a very significant risk. I have to say in answer to that. I was very determined to understand the darkness of my childhood and to have a go at it.”*

Another interviewee (four) had sought to better understand parental mental illness; their own childhood experiences and the possible risks for their own children developing mental illness. Similarly, to interviewee 13, interviewee four also talked to the children about the potential risks of mental illness for them:

*“They said that mum had chronic paranoid schizophrenia. I got a referral from my doctor and spoke to a psychiatrist myself because of all those years of no answers. I only went twice to understand how the illness worked and the chances of us getting it and my children getting it. The only anger I probably felt was thinking that should mum have had children with the risks of the illness. But maybe they didn’t understand the risks and what’s happened. There really wasn’t a contraceptive or anything so much in those days anyway. There was 10% chance of one of us getting it and then less chance for our children getting it. But I rang a helpline one day and they said if any of the grandkids took drugs then they would maybe have a predisposition to having psychotic episodes. I got off the phone and told my children that if they took drugs they’d be like nanny.”*

Yet some of the interviewees had worries in relation to either their own or their child’s emotional health. Interviewee 1 recalled feeling increasingly concerned about supporting the child’s emotional health, while making reference to being parented:

*“I think because my own parents had been so kind of lax, I suppose.* I *had this idea that with children - all you had to do was be nice to them and meet their needs and they’d be happy children. It just wasn’t like that at all...........I couldn’t quite figure it out. Why my child would be kind of moody. So, toddler tantrums and things I found very difficult. I always wanted to please and to meet their needs. It was like the more I did then the worse the situation became. I had no idea about tantrums and things like that.”*

Another interviewee referred to the early bonding period with a new baby, finding it emotionally challenging, feeling that others expected the parental and child bond to be easy. However, the bonding process was difficult. Experiencing confusion and difficulties with the child’s emotions were highlighted throughout many of the narratives of adult children. Interviewees, both male and female, demonstrated ruminating parenting related worries and concerns on a longer-term basis. Interviewee three emphasized experiences of parenting and difficulties adapting to the differing needs of the children. The interviewee was unsure of the parenting role, despite the older child now being school age:

“*I kind of feel like I’m floundering at the moment. I have to kind of parent them quite differently. I’m realising that as I go along, because they are such different children. What I do for one doesn’t work with the other. So I’m still very much finding my feet.”*

Self-reflection and questioning one’s capacity to parent was evident in the transcripts, as interviewee three remarked:

*“You see I always assess myself by am I doing a good job or not. But I feel like I’m doing a fairly good job of parenting. So I don’t think I’m doing a shocking job. But when I do feel like I’ve done badly, I will just pray and say to the Lord, just give me wisdom please. You know, help me say the right words. If all else fails, just love my children and protect them.”*

The story drew attention to an ongoing self-assessment of parenting which, at times, lead the interviewee to view oneself as doing *“badly”*. Similarly, another interviewee (12) made note of undertaking a self -assessment of parenting, when highlighting that:

“*I just get really upset and yell saying you should be angry at me for being a bad parent; they just laugh at me and tell me to get over it and it is all okay - it doesn’t matter.”*

Additionally, interviewees articulated they lacked an internal parenting framework, as interviewee 1 acknowledged:

*“I do strongly identify with not having any internal model of how to parent. Everything has to be kind of consciously put in place. It was quite different for my partner, who just parents without thinking about it. It’s not a big drama. It’s not always the way I’d like, in a way that I agree with, but it’s quite consistently. It’s just there.”*

Reference to an absence of an internal parenting framework and feeling unsure within their parenting roles were common themes within the study. Interviewees utilised several differing methods to develop individual parenting frameworks. The use of parenting books and related literature was helpful for some people. Interviewee two made an active decision to parent accordingly to books rather than any pre-existing model of parenting developed from being parented. Other people considered the reactions from their children and parented accordingly, as interviewee four drew attention to:

*“So, I’m lucky that my children give me feedback. But a lot of it’s seat of the pants sort of stuff. You don’t know. I don’t know whether - because of how mum was I know even less, but you just sort of hang in there.”*

Other adult children relied on their partners to guide the parenting processes. An interviewee’s narrative strongly featured this approach, when remarking:

*“I sort of have to think about it or my partner has got to belt me on the back of the head, to sort of put me on the right path now and again.........I suppose that’s probably what saves me. But I’m not a single parent and I think that’s what probably makes life a lot easier, that it’s us bringing up the kids, just not me, because if it was probably up to me alone they would be in trouble. But because there are both of us there in a loving relationship supporting the kids, it probably does make it a lot easier because - I make mistakes.”*

However, some interviewees parented in direct opposition to the methods of their own parents. Both interviewee six and seven demonstrated this approach of oppositional parenting. Interviewee six highlighted:

*“I would say that my parenting role, I did everything in my power not to be like my parent.”*

While the story of interviewee seven featured a greater level of expressed emotion, the notion of oppositional parenting was clearly identifiable:

“W*hen I come back to how do I want to define myself as a parent, it’s like the exact opposite of what they were, because I don’t want my child ever to look at me and feel ashamed of me or embarrassed of me.”*

Emotional expressions were identified within the narratives particularly when adult children talked about their relationships with their own children. Adult children seeking emotional connectivity with their children and others was established as a sub-theme.

### Adult children seeking emotional connectivity with their children and others

Eleven of the 13 interviewees highlighted their wishes that their children felt loved by them as parents and had a sense of belonging to those around them. Interviewee eight offered a construct about a parent’s love for their children:

*“Loved them desperately and always - that love has been very close....... Security, care, all that. All the needs in life provided. Health - love first, security, care, all the general needs that a child has or the needs of the child. Love is paramount and understanding and facilitating as I said. Facilitating for them to be able to their best and be happy. Not necessarily to succeed with a PhD but to do what they can do that they feel happy about doing, have a good self-image and confidence that they can get on in the world and cope.”*

Adult children universally wanted their children to have a positive sense of self. Positive communications between the adult children and their own children were seen as an important way in which the parent promoted a positive sense of self for their children. Additionally, adult children thought it was important that their children were able to express their own emotions. Interviewee nine noted a desire to provide love for their child in addition to positive hopes of own life-long emotional expression:

*“I think really I just want them to feel comfortable with emotions and to be able to express them to both of us as parents. I sort of give them so much love and cuddles and I can’t get enough cuddles.”*

Adult children reflected an initial positive positioning about their parenting roles. For some, their parenting roles were conceptualised as a vehicle by which they could achieve a greater sense of belonging to others. Several of the interviewees noted that they actively sought belonging to others. Several of the interviewees believed that having a child increased their ease with social interactions and ultimately, their sense of belonging. Interviewee 10 described how becoming a parent facilitated a greater sense of personal connectiveness to others:

*“I feel like coming to this country I’ve been able to reinvent myself. Sort of try to lay to rest all that negativity because my mother was an extremely negative person. She never said I love you, she never said I’m sorry. I just wanted to be in this country - because I was a mother and I had a baby. Whereas it was hard for me to make friends alone, there was something that connected me to other people and so that was a great way for me to become part of this society.”*

Other narratives highlighted that adult children established a greater sense of self-worth from their parenting roles. Positive comments offered by their children about the adult children, noting their children’s achievements or highlighting the positive attributes of their child / children all contributed to their increasing self-worth, as interviewee 10 made reference to:

*“I’m very open with my children, I’m overly loving to them. I treat my children with respect and all these things I show, I get back from them as well. We’ve got a really nice balanced, respectful relationship.”*

Interviewee 11 highlighted comments from the children which provided positive support.

*“My son always says that I’m the best parent ever, the best mother ever. I suppose when he sees his friends’ mothers - because I suppose I’m upbeat with the kids, but sometimes I think maybe that’s just an act. Sometimes I feel a bit more miserable, and it’s like oh, don’t cotton on, you’re miserable, that sort of thing, maybe.”*

Comments from the children positively challenged interviewee 11’s self- beliefs of “*I’m boring, I’m daggy”.* The narrative further highlighted a common discourse that was used by adult children of parents with mental illness surrounding their parenting role. Many interviewees made reference to their parenting in terms of their individual capacity. Positive judgements such as “good”, “success”, “best” and “good” were common place within the narratives. The findings strongly suggested that the adult children’s positioning of parenting was framed within a socially constructed notion of parenting at polarised positions of ‘good’ and ‘bad’.

## Discussions

Adult children in this study reported ongoing anxieties within their parenting roles, regardless of the age of their children suggesting a longitudinal nature to their experiences. The study sought interviewees who had not had any past contact with mental health services or past / current mental health treatments. However, the study did not seek details of possible pre-existing anxiety or other mental health concerns. Given this, interviewee anxiety may be a pre-existing experience prior to becoming a parent. An earlier study [5] found that children sought greater information about their parent’s experiences of mental illness. Previously reported findings from the study with adult children presented in this paper, identified similar findings that children had difficulties accessing information about their parent’s illness due to stigma, which generated fear and mistrust of family and others [14, 15]. Given this, parenting anxiety noted with adult children in this study may be longer standing from their own childhood, as opposed to explicitly related to parenting. Additionally, Petrowski and Stein, [9] noted parentification with an increased sense of care giving and responsibility for both children and adult children of parents with mental illness. This could be a worthy explanation to consider in light of findings which suggested parenting anxieties. The increased sense of responsibility may continue into adulthood, into one’s own parenting role, generating a greater intensity of anxiety.

Adult children’s reflections regarding the quality of relationship with their parent was a significant finding from this study. Previously published findings have centred around loss of self [16] and dehumanisation of a parent [17]. Tensions within the parental and child relationship, when a parent experiences mental illness was a consistent finding in other studies [5, 9]. Petrowski and Stein [9] met with 10 women aged between 18 and 21 years old. Interviewees engaged in semi structured interviews about their experiences of care giving and role reversal. They reported feeling obligated to support their mother with mental illness. Half (five) of the interviewees highlighted a ‘negative’ relationship with their mother. They described feelings of resentment towards their mother. Further, earlier work [28] found that children whose foster mothers who were more accepting of them earlier within the parental and child relationship, had more positive representations of themselves. Both studies [9, 28] reinforced the complex relational interface between children, adult children and parents which was also found in this particular narrative study with adult children. Several of the interviewees in the study presented in this paper reported longer term tensions within their relationships with their parents. Unresolved communications and relationship issues with their parent may be reflected within their relationship with their own children, generating a complex situation of living with rejection of one relationship while establishing an emotional bonding with their own children. This may be a further explanation to the longevity of parenting anxiety for adult children who have lived with a parent with mental illness.

Findings from this study are consistent with previous studies regarding children and adult children’s hypersensitivity to and for parental emotions, when their parent presented with mental illness [1, 29]. However, this study extended this knowledge to suggest that adult children who had lived with childhood parental mental illness, also experienced a similar hypersensitivity to their own children’s emotions, possibly resulting in their own parenting anxiety. Alternatively, adult children’s pre-existing anxiety may result in emotional hypersensitivity towards their own children. Increased adult children’s anxiety within their parenting roles, further demonstrated the complexities of family relationships. Given the relational difficulties noted between parents with mental illness and their children in earlier work [6], it may take the adult child a renewed effort to engage with their own children. Alternatively, this study also found adult children experienced a loss of sense of self [16]. They felt unsure of who they were and of their own emotions. Interviewees reported making active efforts to find themselves to move along a recovery trajectory, alluding to the fact that establishing a sense of self may be integral in order to be able to form an emotional bond with other, including their own children.

One of the study findings highlighted adult children’s worries about their own children developing mental illness. There may be associations between observing for parental symptoms of mental illness during their childhood and later anxieties about their own children’s possible mental illness. This may explain the longitudinal nature of adult children’s parenting anxieties. However, given the stigmas and social values related to mental illness [15], it seems likely that anxiety about potential emergence of familial mental illness may be a common experience for all parents, but at increased intensity for those who already have a family member experiencing mental illness. Despite interviewee’s on-going parenting anxieties in this study, becoming a parent facilitated a sense of recovery for them. This is consistent with previous literature [30] which established a four-stage journey common to families of people experiencing mental illness. The latter stage made reference to increased personal and political advocacy to move towards recovery where a person developed “deepened new meanings and values about themselves, others, their community and larger concerns in their lives” ([30], p., 766). Parenting roles may result in adult children reflecting on their own experiences and values and in doing so, they conceptualise new meanings moving on further towards recovery.

While parenting anxiety may not be unique to those who have experienced childhood parental mental illness, it can have differing manifestations dependent on both the parent’s and child situation. For example: anxiety in relation to childbirth can be a common experience [31], particularly for first time parents who may also have child related and relationship concerns [32]. Additionally, parents may experience increased anxiety when caring for a child with some nature of disability [33, 34]. When analysing maternal feelings within a digital media space, Pedersen and Lupton [35] found 98 of the 100 opening posts presented negative feelings or situations. Interestingly, their study highlighted value loaded questions or statements about one’s parenting role, making reference to the “worst parent in the world” and the emotions of a “good mother” (p. 60). These value loaded and at times, self-critical statements, are very similar to those found in the study presented in this paper with adult children who had experienced parental mental illness. Yet, what seems unique in our study is how the statements by the adult children were accompanied with positivity about their children and a strong desire to share love with them. It may be that adult children who have experienced childhood parental mental illness have a heightened and more conscious awareness of positive engagement with their own children, given their own childhood experiences.

## Conclusions

This paper advanced the knowledge of adult children’s experiences in their own parenting roles after living with childhood parental mental illness. The paper highlighted the need for support for adult children when they become parents to reduce their experiences of anxiety and distress, particularly about their worries about their children developing mental illness. However, the work highlighted adult children’s deep desire for positive relationships with their children which could possibly further their individual recovery.

### Implications for practice

There is a need for all health and social care professionals working with families to consider the longer-term experiences in relation to an individual’s experiences of childhood parental mental illness. Detailed assessment and history gathering is critical to identify adult children who may be at increased risks of parenting anxiety regardless of the age of their children. All health and social professionals have a pinnacle preventative role in supporting families to build resilience and harness positive experiences within familial relationships.

### Study limitations

In order to present integrity of a study, it is important to consider all facets of the research from design, recruitment, analysis and interpretation [25]. It is essential that study limitations are presented alongside some of the decisions which were made throughout the research process [36]. With reference to this particular study with adult children of parents who experienced mental illness, the study design, inclusion and exclusion criteria are worthy of consideration.

The study with adult children who had experienced childhood parental mental illness sought qualitative data. Qualitative studies provide space to explore individual’s experiences and perceptions without pre-determined criteria [37]. Additionally, qualitative studies encourage consideration of experiences within the context of self and relationships [38]. Comparisons between phenomena or population groups are better aligned with quantitative studies [39]. However, the parenting narratives of adults who have not experienced childhood parental mental illness could be helpful to determine if the differing manifestations of parenting anxieties are unique to individuals who have experienced childhood parental mental illness. It seems likely that anxiety in relation to their children developing mental illness is heightened for adults who experienced childhood parental mental illness, but this could also be a concern among many parents regardless of their childhood parenting experiences. The study explicitly sought interviewees who had experienced childhood parental hospitalization for mental illness. Given this, the authors acknowledge that individuals who have experienced childhood parental mental illness, but not parental hospitalization, may have differing experiences of their own parenting journeys. Further, the study inclusion criteria requested that interviewees had not been diagnosed with a mental illness or mental health concern themselves. Adult children who have experienced childhood parental mental illness and diagnosed with mental illness or mental health concerns themselves could present with varied manifestations of parenting anxieties.

The process of data analysis is important to consider when thinking about research integrity. Using software to code qualitative data can reduce researcher bias [40]. Yet researcher bias can still be of concern when using coding software programmes as errors in text prediction are dependent on both the programme and individual users [41]. Data coding and analysis was undertaken by the main author for the adult children study presented in this paper which could have provoked researcher bias. However, the researchers did put other strategies in place to ensure integrity of the study findings. Prior to the commencement of the study, the main author recorded her values of mental health / illness and clinical nursing experiences of working with parents experiencing mental illness and their families. It is thought that researchers telling of their experiences early in the research design process can limit the potential for researcher bias [42]. Additionally, in order to limit bias, all members of the research team read the transcripts and there were regular meetings and discussions until everyone agreed with the interpretation of data and study findings.

## Abbreviations

COREQ checklist: (COnsolidated criteria for REporting Qualitative research, 25) is a 32 – item framework which can be used to confirm the integrity of qualitative studies using interviews and focus groups
